# Sirt1 Protects Endothelial Cells against LPS-Induced Barrier Dysfunction

**DOI:** 10.1155/2017/4082102

**Published:** 2017-10-25

**Authors:** Weijin Zhang, Yaoyuan Zhang, Xiaohua Guo, Zhenhua Zeng, Jie Wu, Yanan Liu, Jing He, Ruiting Wang, Qiaobing Huang, Zhongqing Chen

**Affiliations:** ^1^Department of Critical Care Medicine, Nanfang Hospital, Southern Medical University, Guangzhou 510515, China; ^2^Guangdong Key Lab of Shock and Microcirculation Research, Department of Pathophysiology, Southern Medical University, Guangzhou 510515, China

## Abstract

Sepsis is a threatening health problem and characterized by microvascular dysfunction. In this study, we verified that LPS caused the downregulation of Sirt1 and the hyperpermeability of endothelial cells. Inhibition of Sirt1 with ex527 or Sirt1 siRNA displayed a higher permeability, while activation of Sirt1 with SRT1720 reversed the LPS-induced hyperpermeability, formation of fiber stress, and disruption of VE-cadherin distribution. In pulmonary microvascular vein endothelial cells isolated from wild-type mice, Sirt1 was attenuated upon LPS, while Sirt1 was preserved in a receptor of advanced glycation end product-knockout mice. The RAGE antibody could also diminish the downregulation and ubiquitination of Sirt1 in LPS-exposed human umbilical vein endothelial cells. An LPS-induced decrease in Sirt1 activity was attenuated by the RAGE antibody and TLR4 inhibitor. *In vivo* study also demonstrated the attenuating role of Sirt1 and RAGE knockout in LPS-induced increases in dextran leakage of mesenteric venules. Furthermore, activation of Sirt1 prevented LPS-induced decreases in the activity and expression of superoxide dismutase 2, as well as the increases in NADPH oxidase 4 and reactive oxygen species, while inhibition of Sirt1 aggravated the SOD2 decline. It also demonstrated that Sirt1-deacetylated p53 is required for p53 inactivation, which reversed the downregulation of *β*-catenin caused by LPS.

## 1. Introduction

Sepsis is a fatal health problem and characterized by its complicated infectious pathophysiology [1], variable clinical manifestation [2], high therapeutic cost, and poor eventual prognosis [3]. Sepsis is posing great threat to our health, but we still have known little in the understanding of the occurrence and treatment of sepsis. Though sepsis is elusive, we are gradually getting to realize the participation of microvascular barrier disruption and vascular dysfunction in the pathogenesis and development of sepsis. Vascular endothelial cells (ECs) lining the intima of blood vessels are critical in the regulation of nutrient trafficking and maintenance of microvascular homeostasis. Disruption of the EC barrier leads to vascular hyperpermeability and leakage of albumin and fluid, resulting in tissue edema and potential occurrence of organ dysfunction [4]. A great number of compelling evidences reveal that endothelial dysfunction acts as one of the mechanisms underlying sepsis and preventing vascular extravasation could ameliorate mortality from sepsis through the reinforce of endothelial cytoskeleton and modulation of endothelial junctions [5, 6]. Sepsis is known to induce acute lung injury accompanied by increased pulmonary microvascular leakage and wet/dry weight ratio [7], hinting that the EC barrier might be a promising approach for more profound understanding of sepsis. Nevertheless, the understanding of the molecular mechanisms remains to be explored.

It is acknowledged that the silent information regulator 2 (SIR2) complex is composed of 4 groups, including group I with sirtuin 1 (Sirt1), 2, and 3; group II with Sirt4; group III with Sirt5; and group IV with Sirt6 and 7 [8]. By far, Sirt1 has been the most widely studied and reported. As a nicotinamide adenine dinucleotide- (NAD^+^-) dependent histone deacetylase, Sirt1 functions as a master regulator of ageing, apoptosis, and stress response [9, 10]. Sirt1 is known to regulate EC functions by targeting a broad spectrum of its substrates, including p53 [11], p66Shc [12], and eNOs [13]. Furthermore, Sirt1 was also demonstrated to have a pivotal role in the defense of inflammation in lipopolysaccharide- (LPS-) induced acute lung injury (ALI). Inhibition of Sirt1 with Sirt1 siRNA diminished the salutary effect of resveratrol on inflammation resistance [14]. Deficiency of Sirt1 increases microvascular inflammation, morbidity, and mortality in early sepsis, whereas the Sirt1 activator reversed the aforementioned effect, indicating that Sirt1 may play a protective role in the development of sepsis [15]. However, these data did not clarify whether Sirt1 is beneficial to the EC barrier. If so, the molecular mechanism in which Sirt1 exerts the protective role in LPS-induced EC dysfunction also needs to be unveiled. The purpose of this research was to study whether and how Sirt1 affects LPS-sparked EC barrier disruption. Together with this, we also deem Sirt1 as a node of the event through enhancing superoxide dismutase 2 (SOD2) expression and inhibiting NADPH oxidase 4 (NOX4) expression to resist oxidative stress and inactivating p53 to inhibit the loss of junctional protein *β*-catenin.

SOD2 is well known to scavenge reactive oxygen species (ROS) and ameliorate ROS-induced EC hyperpermeability [16]. The interplay between Sirt1 and SOD2 has been reported to increase ROS resistance in ECs [17–19]. It was also confirmed that Sirt1 inhibition was engaged in upregulation of NOX4, eventually leading to endothelial dysfunction due to O_2_^•−^ production [20]. Based on these findings, we hypothesized that Sirt1 could protect the EC barrier against LPS by the mechanism involving the upregulation of SOD2 and the downregulation of NOX4 to resist ROS generation. Furthermore, the adherence junction (AJ) protein *β*-catenin is of great significance for the EC barrier, and the degradation of *β*-catenin tends to disrupt the barrier [21, 22]. p53 has been demonstrated to be critical in the occurrence of brain edema [23], and inactivation of p53 has the potential to prevent the degradation of *β*-catenin [24]. p53 was first found as the target of Sirt1, and quantities of studies have revealed the role of Sirt1 in deacetylating p53 and its inactivation [25, 26]. Hence, we also speculated that Sirt1-deacetylated p53 could be another promising target for the protection of junction proteins and the EC barrier.

## 2. Results

### 2.1. LPS Increases Hyperpermeability in HUVECs

**Figure 1:**
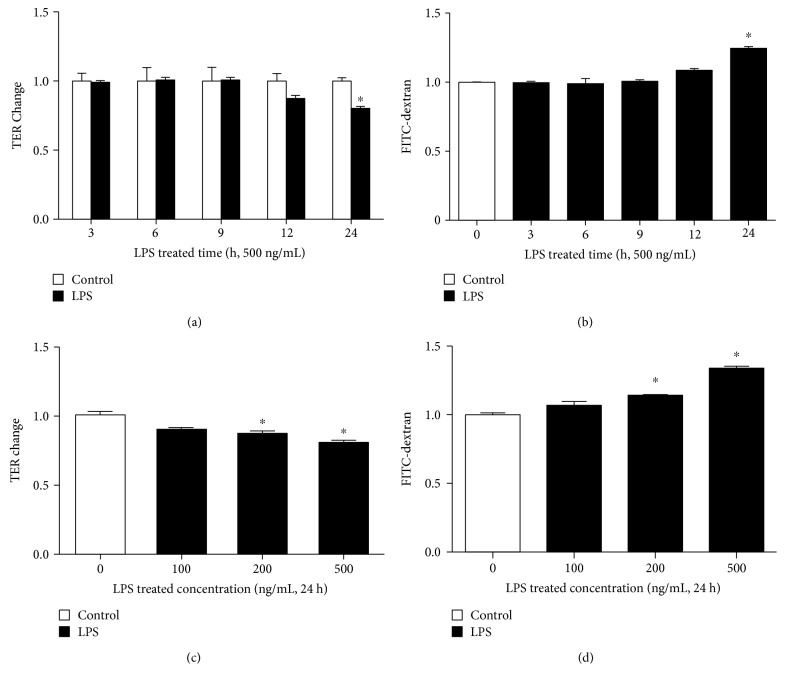
Effects of LPS on EC permeability. Cells were (a, b) treated with 500 ng/mL of LPS for 3, 6, 9, 12, and 24 h or with LPS (c, d) at 100, 200, and 500 ng/mL for 24 h, compared to the control with the culture medium. Permeability was assessed by TER and flux of FITC-dextran. *n* = 3 per group. ^∗^*P* < 0.05 versus control.

We incubated human umbilical vein endothelial cells (HUVECs) with LPS at different concentrations for various durations. Permeability coefficient was measured by transendothelial electric resistance (TER) and flux of FITC-dextran. As shown in Figure 1(a), 500 ng/mL LPS increased EC permeability in a time-dependent manner. We found that the TER level was gradually decreased from 12 h, and it did not become significant until 24 h, which coincided with the FITC-dextran coefficient (Figure 1(b)). Therefore, we used 24 h as the proper time for LPS-induced barrier dysfunction. Accordingly, we found that LPS induced EC hyperpermeability for 24 h in a dose-dependent fashion. An increase in FITC-dextran flux was observed from 100 ng/mL, and the change was significant at 200 and 500 ng/mL, consistent with TER measurement (Figures 1(c) and 1(d)). Hence, ECs were treated with 500 ng/mL LPS for 24 h in the following barrier function detections.

### 2.2. Sirt1 Protects ECs from LPS-Evoked Hyperpermeability

**Figure 2:**
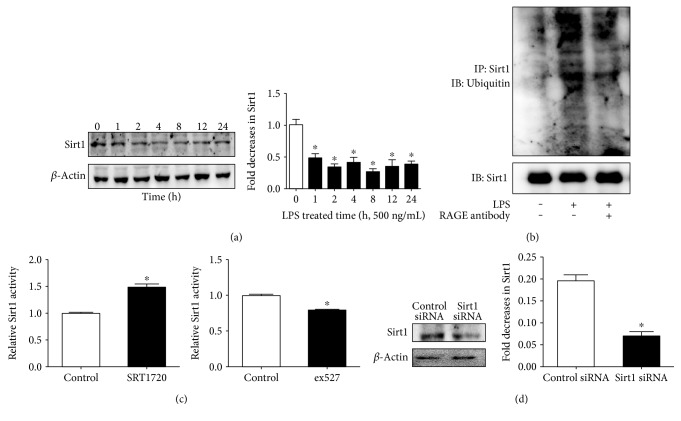
Protein expression of Sirt1 and activity changes in HUVECs. (a) HUVECs were exposed to 500 ng/mL LPS for 1, 2, 4, 8, 12, and 24 h, and protein expression of Sirt1 was detected by WB. 0 h was used as the control. (b) Cells were stimulated with or without 500 ng/mL LPS for 1 h in the presence or absence of the RAGE antibody (10 *μ*g/mL, 1 h). The immunoprecipitates were analyzed with anti-Sirt1 and anti-ubiquitin antibodies. (c) Sirt1 activity was examined and SRT1720 enhanced Sirt1 activity, while ex527 inhibited Sirt1 activity. (d) ECs were treated with either control siRNA or Sirt1 siRNA targeting Sirt1. The effect of siRNA on the Sirt1 protein expression was measured by WB. *n* = 3 per group. ^∗^*P* < 0.05 versus control or control siRNA.

Next, we explored whether protein expression of Sirt1 was changed after exposure to LPS. As shown in Figure 2(a), treatment of 500 ng/mL LPS induced a sharp reduction in Sirt1 expression and it kept at a low level ranging from 1 h to 24 h, indicating the critical role of Sirt1 in LPS-evoked EC reaction. Next, the result showed that Sirt1 ubiquitination was increased after LPS treatment, suggesting that ubiquitination was responsible for the LPS-induced Sirt1 decrease (Figure 2(b)). To evaluate the role of Sirt1 in LPS-induced hyperpermeability, we firstly examined the influence of the Sirt1 activator SRT1720 and the Sirt1 inhibitor ex527 on Sirt1 activity, as well as the effect of Sirt1 siRNA on Sirt1 protein expression.  Figure 2(c) shows a significant increase in Sirt1 activity in HUVECs pretreated with SRT1720 (5 *μ*M, 24 h) and a decrease in Sirt1 activity in HUVECs pretreated with ex527 (20 *μ*M, 1 h). Then, Sirt1 siRNA was applied, and the result showed that Sirt1 siRNA (20 *μ*M) efficiently suppressed the protein expression of Sirt1 (Figure 2(d)).

**Figure 3:**
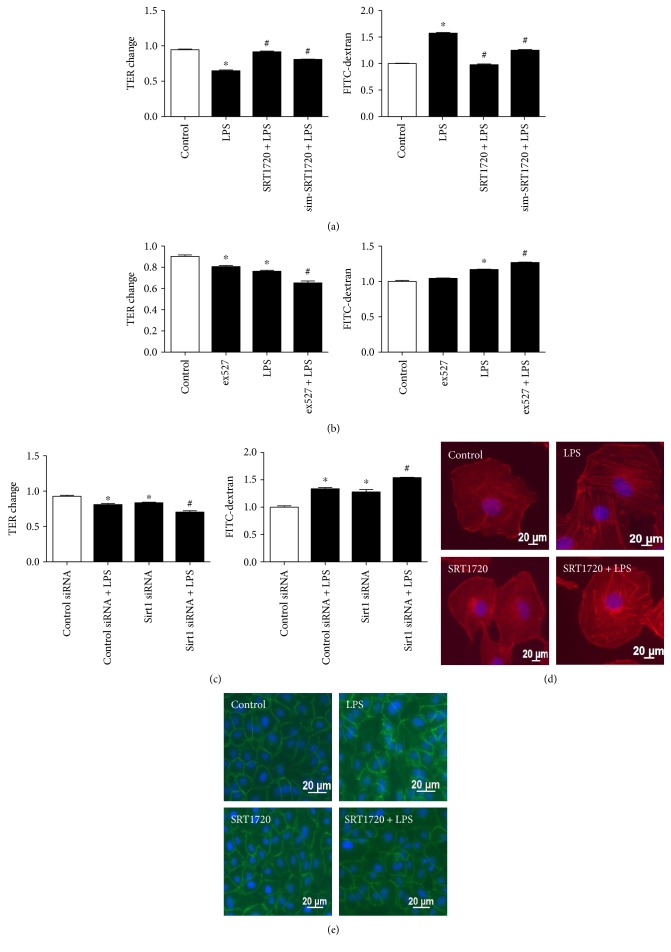
Protective effect of Sirt1 on LPS-induced EC hyperpermeability. (a) SRT1720 prevented LPS-induced EC hyperpermeability. HUVECs were pretreated with SRT1720 (5 *μ*M) 24 h before (SRT1720 + LPS) or at the time (sim-SRT1720 + LPS) of LPS (500 ng/mL, 24 h) treatment; then, permeability of monolayers was measured. (b) ex527 exacerbated LPS-evoked EC hyperpermeability. HUVECs were pretreated with ex527 (20 *μ*M) 1 h before LPS (500 ng/mL, 24 h) treatment; then, permeability of monolayers was measured. (c) Sirt1 siRNA increased LPS-evoked EC hyperpermeability. Cells treated with control or Sirt1 siRNA were exposed to LPS (500 ng/mL, 24 h); then, permeability of monolayers was measured. *n* = 3 per group. ^∗^*P* < 0.05 versus control or control siRNA, ^#^*P* < 0.05 versus LPS or control siRNA + LPS. (d-e) The effect of SRT1720 on the distribution of F-actin and VE-cadherin. Cells were pretreated with SRT1720, followed by examining F-actin and VE-cadherin using confocal microscopy.

The protective role of Sirt1 in LPS-induced EC hyperpermeability was then verified by monitoring the monolayer barrier and detecting the morphological alterations of F-actin and VE-cadherin. It was revealed that the decrease in TER level induced by LPS was remarkably reversed by both pretreatment and simultaneous treatment with SRT1720, consistent with the reversed decrease in flux of dextran (Figure 3(a)). To confirm the important role of Sirt1, the Sirt1 inhibitor and siRNA were also applied. Ex527 and Sirt1 siRNA could further increase EC permeability, indicating the deteriorating EC barrier for the lack of Sirt1 activity (Figures 3(b) and 3(c)). Afterwards, distribution of F-actin and VE-cadherin was observed. The quiescent cells showed F-actin in a normal condition, characterized by typical and intact outline of the cytoskeleton. LPS treatment caused a redistribution of F-actin with stress fiber formation, which rendered cells to contract towards the center of the cell. However, the formation of stress fiber was attenuated in the application of SRT1720 (Figure 3(d)). Consistently, in response to LPS, VE-cadherin was dissociated and disrupted, which was also reversed by SRT1720 pretreatment (Figure 3(e)).

**Figure 4:**
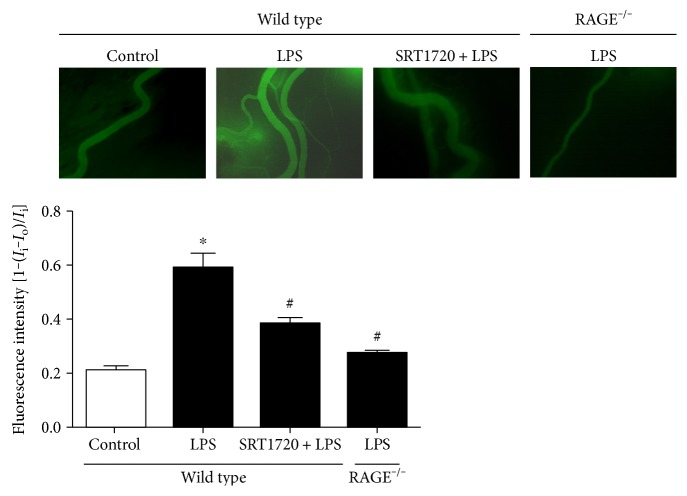
Role of Sirt1 and RAGE in microvascular hyperpermeability induced by LPS. Mice were pretreated with SRT1720 or saline via tail vein injection 2 h prior to LPS treatment. The mice in the LPS group were injected with 15 mg/kg LPS intraperitoneally, followed by carotid vein cannulation and exudation measurement 6 h later. Microvascular permeability was determined by the relative dextran density outside the vessels to that inside the vessels. *n* = 3. ^∗^*P* < 0.05 versus control, ^#^*P* < 0.05 versus wild type with LPS treatment.

*In vivo* (Figure 4), images from microscopy showed little extravasation of dextran in the saline-treated mice. By contrast, the LPS-injected mice showed a remarkable increase in dextran flux outside the vessels, implying the microvascular hyperpermeability caused by LPS. However, pretreatment of SRT1720 could significantly attenuate the leakage. All these *in vivo* and *in vitro* data suggest that Sirt1 played a pivotal role in EC hyperpermeability in response to LPS.

### 2.3. RAGE and TLR4 Are Required for LPS-Induced Sirt1 Downregulation

**Figure 5:**
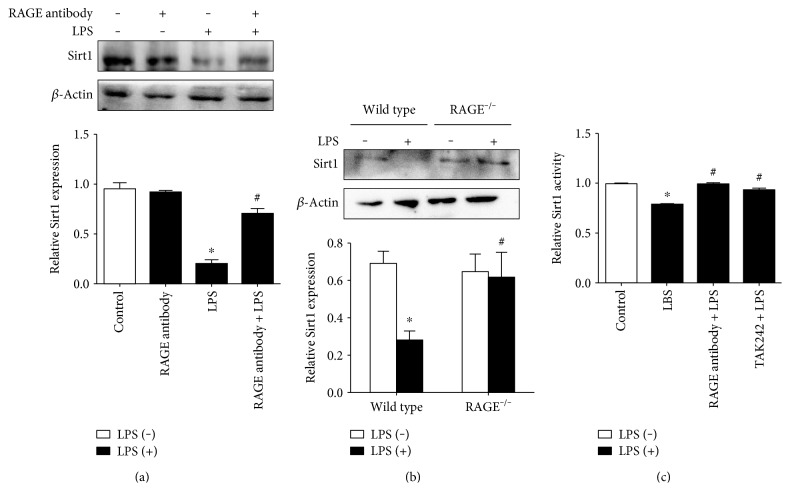
Involvement of RAGE and TLR4 in the LPS-induced Sirt1 decrease in ECs. (a) Pretreatment of the RAGE antibody (10 *μ*g/mL) for 1 h attenuated Sirt1 decreases in HUVECs. (b) Knockout of RAGE attenuated the LPS-induced Sirt1 decrease in PMVECs. PMVECs of knockout mice and wild-type mice were incubated with or without LPS. Expression of Sirt1 was evaluated by WB. (c) Pretreatment of the RAGE antibody (10 *μ*g/mL) and TLR4 inhibitor (3 *μ*M) for 1 h attenuated the LPS-induced decrease in Sirt1 activity in HUVECs. *n* = 3. ^∗^*P* < 0.05 versus control or wild type without LPS treatment, ^#^*P* < 0.05 versus LPS or wild type with LPS treatment.

After the critical role of Sirt1 in LPS-induced EC barrier disruption was confirmed, the effect of RAGE on the LPS-evoked Sirt1 decrease was evaluated. *In vitro*, the RAGE antibody was shown to abolish the increase in Sirt1 ubiquitin and the decrease in Sirt1 expression in HUVECs (Figures 2(b) and 5(a)). In pulmonary microvascular vein endothelial cell (PMVECs) of wild-type mice, the expression of Sirt1 was attenuated in response to LPS, while the decrease in Sirt1 was reversed in RAGE-knockout mice (Figure 5(b)). Furthermore, the RAGE antibody and the TLR4 inhibitor TAK242 reversed the LPS-induced decrease in Sirt1 activity. These data revealed that RAGE and TLR4 were required for the Sirt1 decline concomitantly. The *in vivo* study also showed that the exudation of dextran from mesenteric venules was decreased in RAGE^−/−^ mice after LPS administration (Figure 4). All these suggested that RAGE and TLR4 were essential for the LPS-sparked Sirt1 decrease and the subsequent hyperpermeability.

### 2.4. Sirt1 Reverses the Decrease in SOD2 Level and the Increase in NOX4 Level to Resist ROS Induced by LPS

**Figure 6:**
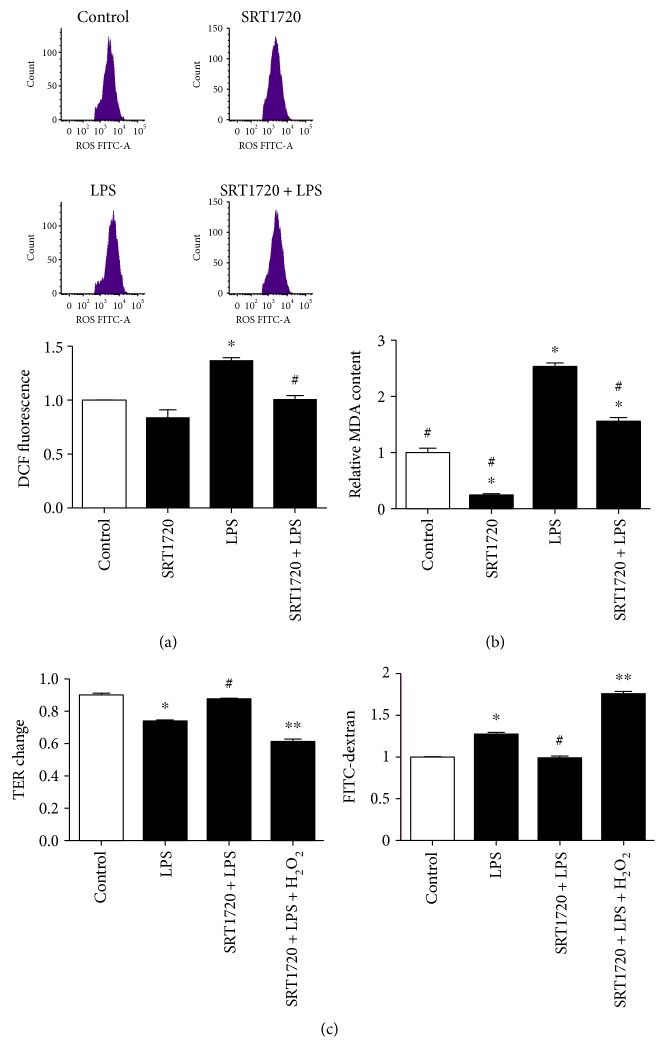
Effect of Sirt1 on the alteration of MDA, as well as on ROS generation in HUVECs. (a) SRT1720 abolished LPS-induced ROS generation. (b) SRT1720 attenuated LPS-induced MDA elevation. (c) H_2_O_2_ abolished the protective effect of SRT1720 on EC permeability. HUVECs were pretreated with or without SRT1720, then exposed to the control medium or LPS. Cells in the group SRT1720 + LPS + H_2_O_2_ were followed by 100 mM H_2_O_2_ treatment for 15 min. *n* = 3^∗^*P* < 0.05 versus control or control siRNA, ^#^*P* < 0.05 versus LPS or control siRNA + LPS, and ^∗∗^*P* < 0.05 versus SRT1720 + LPS.

Firstly, we determined the ROS level following LPS (500 ng/mL, 6 h) treatment by using peroxide-sensitive dye H_2_DCF-DA. As shown in Figure 6(a), ROS level was significantly enhanced compared to that in the control group, whereas this increase was notably abolished by pretreatment of SRT1720. Together, MDA level was assessed and it was increased in the LPS group, which was reversed in the SRT1720 + LPS group (Figure 6(b)). As H_2_O_2_ could generate more ROS and antagonize the protective effect of SOD2 on ROS decreases [27], H_2_O_2_ was applied to verify the role of SOD2 in the EC barrier.  Figure 6(c) showed that H_2_O_2_ treatment remarkably reversed the protective effect of SRT1720 on LPS-induced barrier disruption.

**Figure 7:**
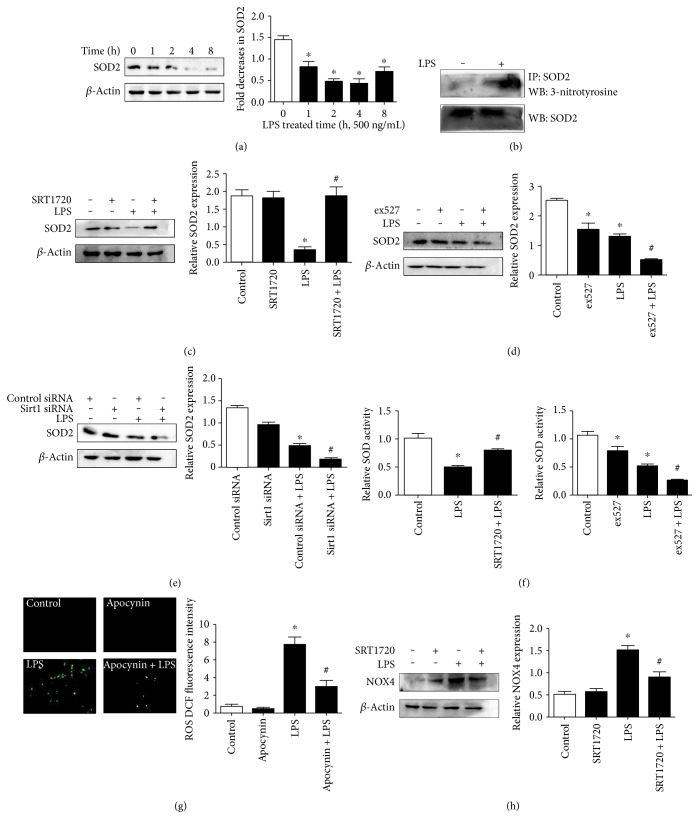
Effect of Sirt1 on the alteration of protein expression and activity of SOD2, as well as on NOX4 generation in HUVECs. (a) LPS evoked the decline in SOD2 expression in a time-dependent manner. (b) LPS induced tyrosine nitration of SOD2. (c–e) SRT1720 abolished the decrease in SOD2 expression upon LPS stimulation while ex527, as well as Sirt1 siRNA, duplicated the effect. (f) The Sirt1 activator reversed the LPS-induced decrease in SOD activity. ECs were treated with or without SRT1720 or ex527, followed by treatment of LPS (500 ng/mL, 1 h); then, SOD activity was detected using an SOD assay kit. (g) The NOX4 inhibitor apocynin (300 *μ*mol/L, 1 h) attenuated LPS-induced ROS generation. ROS was measured through a fluorescent microscope. (h) SRT1720 abolished LPS-induced NOX4 elevation. *n* = 3. ^∗^*P* < 0.05 versus control or control siRNA, ^#^*P* < 0.05 versus LPS or control siRNA + LPS.

To elucidate the signal pathways that may respond to Sirt1 activation, HUVECs were stimulated with LPS to detect SOD2 expression. Treatment of 500 ng/mL LPS significantly caused a sharp decrease in SOD2 expression at 1 h, reaching the bottom at 4 h and slightly mounting at 8 h (Figure 7(a)). It was shown that tyrosine nitration of SOD2 was induced by LPS compared with control, leading to SOD2 inactivation (Figure 7(b)). Furthermore, pretreatment of the Sirt1 activator SRT1720 alleviated the decrease in SOD2, while inhibition of Sirt1 with ex527 and siRNA aggravated the SOD2 decline (Figures 7(c), 7(d), and 7(e)), which were consistent with the results of SOD activity in different interventions (Figure 7(f)). Then, the NOX inhibitor apocynin was applied to verify the role of NOX in LPS-sparked ROS generation.  Figure 7(g) shows that apocynin diminished ROS elevation induced by LPS, indicating NOX as the source of ROS. Pretreatment of SRT1720 alleviated the increase in NOX4 induced by LPS, strengthening the probability that Sirt1 may preserve the EC barrier through enhancing SOD2 expression and attenuating NOX4 level together to resist ROS generation.

### 2.5. The Activation of Sirt1 and the Inhibition of p53 Prevented the LPS-Evoked Decrease in *β*-Catenin Expression

**Figure 8:**
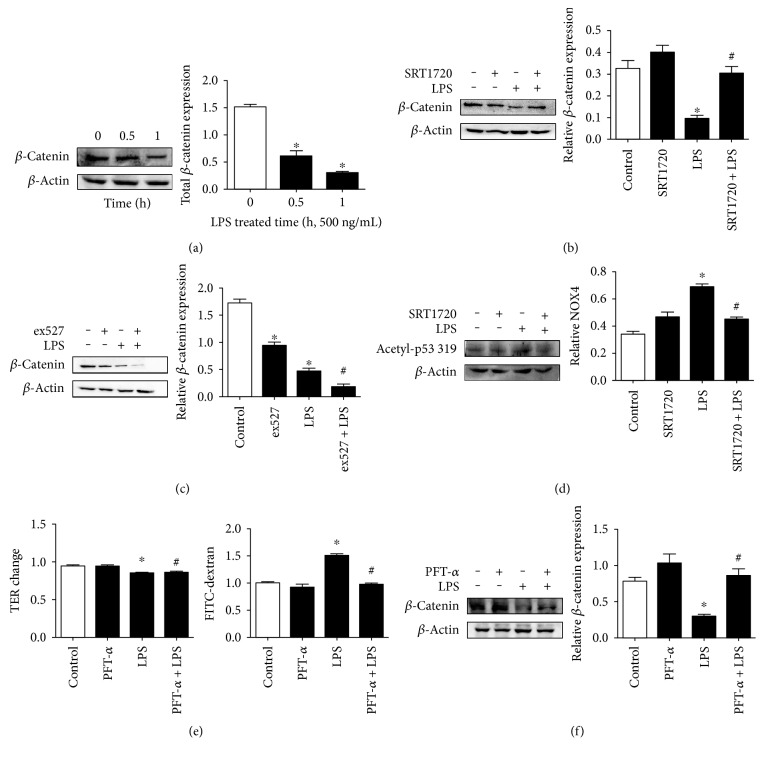
The role of SRT1720 and p53 in the downregulation of *β*-catenin induced by LPS in HUVECs. (a) Time-dependent changes in expression of *β*-catenin induced by 500 ng/mL LPS. (b) Pretreatment of SRT1720 prevented LPS-induced *β*-catenin downregulation. (c) Pretreatment of ex527 aggravated LPS-induced *β*-catenin downregulation. (d) SRT1720 attenuated LPS-evoked p53 319 acetylation. (e) p53 inhibition with PFT-*α* prevented LPS-induced EC hyperpermeability. (f) p53 inhibition with PFT-*α* prevented the LPS-induced decrease in *β*-catenin expression. *n* = 3^∗^*P* < 0.05 versus control, ^#^*P* < 0.05 versus LPS.

It has been shown that Sirt1 deacetylates and inactivates p53. The p53 inactivation could prevent the degradation of *β*-catenin [24]. However, it remains unknown whether there is a relationship between Sirt1 and *β*-catenin in a sepsis model. Firstly, to observe the influence of LPS on the change of AJ protein, 500 ng/mL LPS was exposed to ECs. The time-response experiment (Figure 8(a)) showed a gradual decrease in *β*-catenin expression from 0.5 h to 1 h, indicating that LPS might cause EC hyperpermeability via the reduction in adherent junction proteins. However, downregulation of *β*-catenin was abolished due to the application of SRT1720, while pretreatment of ex527 aggravated the downregulation of *β*-catenin (Figures 8(b) and 8(c)), which coincided with the effect of SRT1720 and ex527 on LPS-induced barrier disruption (Figures 3(a) and 3(b)). As p53 inhibition has been shown to prevent *β*-catenin degradation and p53 was a typical downstream of Sirt1 [25], we next examined the possible role of Sirt1-deacetylated p53 in the increased expression of *β*-catenin.  Figure 8(d) shows that acetyl-p53 319 was increased in response to 500 ng/mL LPS for 1 h. However, the acetylated level was attenuated after SRT1720 addition. Significance was not observed concerning total p53 (Supplementary Figure 1 in Supplementary Material available online at https://doi.org/10.1155/2017/4082102). The pretreatment of HUVECs with the p53 inhibitor PFT-*α* (30 *μ*M) [28] for 24 h could also reverse barrier dysfunction induced by LPS in both TER level and the flux of FITC-dextran, implying the important role of p53 in EC hyperpermeability after LPS treatment (Figure 8(e)). Next, PFT-*α* was used to further specify the role of p53 in *β*-catenin expression. The data showed that the blockade of p53 abolished the reduction in *β*-catenin (Figure 8(f)). All these data suggest that Sirt1-deacetylated p53 was probably engaged in the preservation of *β*-catenin expression to mitigate EC hyperpermeability induced by LPS.

## 3. Discussion

Sepsis represents a lethal health issue, not only relating to the complicated pathogenesis but also resulting in the prevalence of organ dysfunction and mortality. Though our realization of sepsis is ambiguous, we are still making progress in exploring it step by step. The development of sepsis is linked with hemodynamic alternations and microvascular barrier dysfunction to some degree. The disruption of vascular endothelial integrity results in capillary leakage and the loss of fluid. However, the mechanism underlying EC dysfunction caused by sepsis is still not clear. Our goal was to probe the relationship of LPS and the specific molecule Sirt1 in manipulating the function of the endothelial barrier.

As an NAD^+^-dependent deacetylase, the longevity regulator Sirt1 has gained great attention for its salutary effect on vascular function. Accumulating evidences have revealed that Sirt1 is connected closely with vascular disease and contributes to the modulation of key metabolic processes, including vascular ageing [29], ROS resistance [30], and apoptosis [31]. The present study revealed that Sirt1 protein was dramatically suppressed and sustained at a low level until 24 h in the application of LPS. It is ubiquitin that mediates a Sirt1 decrease. Substantial evidence shows that extreme stress response to sepsis induces remarkable drop of Sirt1 expression and hyperinflammation. Resolution of acute inflammation may rebalance Sirt1 and restore homeostasis [32]. Perhaps, ECs might firstly undergo a short time of Sirt1 inhibition in the early stage, and Sirt1 expression might be increased to defend inflammation and to keep homeostasis later. However, elevation of Sirt1 was not observed prior to 24 h. Perhaps, prolonged time for stimulation should be conducted. In contrast, other studies showed that Sirt1 protein was elevated in human gingival fibroblasts [33]. There is no consensus on how Sirt1 was changed upon stress. The difference of Sirt1 response to LPS may be explained by different cell types and different conditions of LPS treatment. The mechanism and the regulation process are very complex, and further study remains to be explored. Given that Sirt1 exerts a protective effect on ECs, we speculated that Sirt1 might also ameliorate LPS-induced barrier dysfunction. In our study, we have demonstrated that LPS sparked EC hyperpermeability in a dose- and time-dependent manner. Inhibition of Sirt1 activity with a specific inhibitor or siRNA aggravated the barrier dysfunction, accompanied by F-actin rearrangement and VE-cadherin disruption, while elevation of its activity with an activator reversed the EC hyperpermeability, indicating the protective role of Sirt1 in LPS-induced EC dysfunction. Furthermore, treatment of LPS and Sirt1 activator at the same time could also reverse the hyperpermeability, indicating its remedial role in the EC barrier. Loss of the AJ protein *β*-catenin caused by LPS was also reversed due to upregulation of Sirt1 activity. Furthermore, *in vivo* experiment revealed that extravasation of FITC-labelled dextran from mesenteric venules was elevated in LPS-treated mouse, while activation of Sirt1 with its agonist could counteract the exudation. These data illuminate an exciting avenue to further explore the potent molecular mechanism implicated in the beneficial role of Sirt1 in the protection of the EC barrier. And we speculate that Sirt1 might act as a critical node to respond to LPS-RAGE signaling pathways and to trigger the elevation of SOD2 and attenuation of NOX4 to resist ROS and the downregulation of p53 activity to prohibit the loss of the junctional protein, *β*-catenin.

LPS binding to RAGE is widely acknowledged [34, 35]. RAGE plays a pivotal role in mediating mortality after cecal ligation and puncture (CLP), the typical model of sepsis [36]. Interestingly, exogenous soluble RAGE (sRAGE), composed of the extracellular domain of RAGE, could block the response to LPS for its antagonist effect with the intracellular domain of RAGE. Furthermore, elevation of sRAGE was detected in septic patients compared with healthy volunteers, indicating that sRAGE could be a candidate as a new sepsis marker [37]. RAGE is critical in Sirt1 inhibition through the ubiquitin-proteasome pathway in diabetic nephropathy [38]. In the present study, we found that RAGE was engaged in LPS-induced downregulation of Sirt1 expression and activity in HUVECs. RAGE knockout mostly abrogated the LPS-sparked Sirt1 decrease and extravasation of mesenteric venules in a septic mouse model. Nevertheless, our result did not clarify the involvement of Sirt1 in the decrease in capillary leakage in RAGE^−/−^ mice. As we all know, in vivo study was characterized by the complexity of tissues and different cell types, including immune cells and other kinds of cells, which could not explain strongly the exact change of Sirt1 distributed in vessels, especially in the microvasculature. Thus, in our primary study shown in Figure 5(b), we isolated the PMVECs and were convinced that the decrease in Sirt1 expression induced by LPS was reversed in RAGE^−/−^ cells, to some extent, indicating Sirt1 involvement in the decrease in capillary leakage in RAGE^−/−^ mice. Ubiquitin-specific protease 22 (USP22) was reported to be associated with Sirt1 and can decrease Sirt1 ubiquitin. Furthermore, the AGEs-RAGE-USP22-Sirt1 has been elucidated as a cascade pathway that participated in the pathological progression of diabetic nephropathy. We also verified that LPS induced a Sirt1 decrease through ubiquitin pathways. Thus, we speculate that USP22 may also be the downstream of RAGE, which remains to be explored. Regarding other receptors for the ligand LPS, Toll-like receptor 4 (TLR4) was the first found LPS receptor and deficiency of TLR4 abated the LPS signaling pathway [39]. The present study found that a TLR4 inhibitor reversed an LPS-induced decrease in Sirt1 activity. These data revealed that RAGE and TLR4 were required for a decline in Sirt1 activity. To date, multiple studies have shown that RAGE and TLR4 could trigger similar inflammatory pathways in response to microbial products, including LPS [35, 40], high-mobility group protein 1 (HMGB1) [41, 42], and S100A8/A9 [43, 44]. Coactivation of RAGE and TLR4 was required to induce cellular responses when LPS was in complex with HMGB1 [45]. However, most evidences were focused on the downstream of RAGE and TLR4, and little was known about the crosstalk between them. It remains obscure whether RAGE and TLR4 combine with each other after LPS exposure. Both RAGE and TLR4 are comprised of three domains, including the extracellular domain, transmembrane domain, and intracellular domain. Perhaps, the structural and the biochemical factors of RAGE and TLR4 promote their cointeraction with LPS. Moreover, it is noteworthy that Toll-like receptor 2 (TLR2) serves as another crucial receptor of LPS, but it remains unknown whether TLR2 is engaged in response to LPS *in vivo* [46]. Hence, much more investigation should be warranted involving the potent role of TLR2 in an LPS-induced Sirt1 decline and EC hyperpermeability.

Growing evidence has proven that ROS render EC dysfunction, characterized by increasing fluid flux and inflammation reaction [47, 48]. Along with this, LPS-induced oxidative stress is closely related to lung vascular hyperpermeability and acute lung injury [49]. As a notable pioneer for scavenging ROS, SOD2 has been elucidated to contribute greatly to oxidative stress defense for decades [50]. As expected, we verified that the level and activity of SOD2 were remarkably decreased after exposure to LPS, consistent with the increase in ROS fluorescence by flow cytometry. However, the aforementioned effect was reversed by the upregulation of Sirt1, indicating Sirt1 as the activator of SOD2 to resist LPS-evoked ROS generation. In agreement with these findings, our previous research also revealed that the enhanced Sirt1 activity suppressed ROS by increasing expression of SOD2 to prevent hepatocyte mitochondrial dysfunction following hemorrhagic shock [51]. Furthermore, Sirt1 could increase transcription of SOD via deacetylating FOXO3a, hinting that the Sirt1/FOXO/SOD pathway may contribute to the suppression of ROS [17]. Another pathway, Sirt1-peroxisome proliferator-activated receptor gamma coactivator (PGC-1*α*), is also potent to trigger SOD2 expression. Nevertheless, not only is the increase in SOD2 responsible for the reduced oxidative stress but the decrease in NOX4 is also critical in the suppression of ROS sources for the beneficial effect of Sirt1. Our study proved that it was the case. This was supported by the downregulation of NOX4 and the NOX4-driven ROS production after Sirt1 activation. The mechanism underlying Sirt1-dependent NOX4 inhibition may involve decreased PGC-1*α* acetylation and the subsequent peroxisome proliferator-activated receptor-*α* (PPAR*α*) activation [20]. Hence, our finding illustrates the possible salutary role of Sirt1 in diminishing the LPS-induced ROS generation by elevating the expression and activity of SOD2 and attenuating NOX4 expression. Last but not the least, the mechanism by which Sirt1 upregulates SOD2 and downregulates NOX4 expression in an LPS-treated model still remains obscure. Thus, further exploration needs to be elucidated.

Intracellular junctional proteins have been elucidated to maintain the cell-cell barrier [52, 53]. Degradation of the AJ protein *β*-catenin [54, 55] interferes with the sealing efficiency of ECs and results in barrier disruption. Our study revealed that activation of Sirt1 reversed LPS-sparked loss of *β*-catenin, while inactivation of Sirt1 exacerbated the effect. However, the mechanism was not clear. It has been demonstrated that downregulation of *β*-catenin could be evoked by activated p53 [24]. As p53 was the first found target of Sirt1 and Sirt1 could inactivate p53 through deacetylating it in an LPS-treated model, we next determined whether p53 contributes to the LPS-induced loss of *β*-catenin. Our present study revealed that Sirt1 activation was required for p53 deacetylation and *β*-catenin upregulation. Furthermore, the p53 inhibitor ameliorated the LPS-induced downregulation of junction proteins and EC hyperpermeability, indicating the pivotal role of p53 in LPS-evoked EC dysfunction. Thus, it is likely that Sirt1 takes part in p53 inactivation, which is involved in the prevention of junction protein downregulation.

However, there are some limitations regarding experimental methods and designs. Firstly, we exposed mice to LPS injection intraperitoneally as the septic model. However, sepsis is not merely the event of gram-negative infection, and it refers to a complicated state characterized by a combination of gram-negative and gram-positive infection, involving systemic problems, including problems in the respiratory, urinary, gastrointestinal, and central nervous systems [56]. Hence, our model could not thoroughly explain the potent mechanism underlying sepsis-associated EC dysfunction, while the CLP model could. Nevertheless, at least, we are able to have a better understanding of the specific role played by gram-negative infection. Furthermore, the most crucial reason why we preferred LPS treatment to the CLP model is that CLP operation could probably ignite severe inflammation reaction and rise hyperpermeability of the mesenteric venules, which would arouse controversy surrounding the root cause of hyperpermeability *in vivo*, as to whether the injury was caused by CLP operation or by sepsis. So LPS treatment could avoid the ambiguity.

In summary, our present study revealed that Sirt1, as a hub, responds to LPS-RAGE signaling pathways and attenuates LPS-induced hyperpermeability through diminishing the generation of ROS and reversing the damage of junction proteins. Our finding sheds light on the possible protective role of Sirt1 in LPS-induced EC dysfunction and provides the possibility that Sirt1 could be a potent therapeutic target for the prevention and therapy of LPS-induced EC dysfunction.

## 4. Methods

### 4.1. Cells, Reagents, and Antibodies

HUVECs are purchased from ScienCell, and LPS was acquired from Sigma. SRT1720 (S1129), ex527 (S1514), and PFT-*α* (S2929) were purchased from Selleck (USA). Sirt1 siRNA (sc-40986) and control siRNA (sc-37007) were from Santa Cruz Biotechnology. 2,7-Dichlorofluoresein diacetate (H_2_DCF-DA) was purchased from Sigma (USA). Antibodies against *β*-catenin (ab32572), against VE-cadherin (ab33168), and against SOD2 (ab13533) were from Abcam (USA). An antibody against Sirt1 (A0127) was from Abclonal (Boston, USA). An antibody against acetyl-p53 (K319) (YK0015) was from Immunoway (USA). An antibody against 3-nitrotyrosine (OM265454) was from OmnimAbs (USA). An antibody against p53 (WL01919) was from Wanleibio (China). An antibody against ubiquitin (3936S) was from CST (USA).

### 4.2. Assay for Quantification of Sirt1 Activity

Measurement of Sirt1 activity was performed using a Sirt1 activity assay kit (ab156065, Abcam). Cells were lysed and immunoprecipitated with a Sirt1 antibody (10 *μ*g). Afterwards, the reaction mixture containing 30 *μ*L ddH_2_O, 5 *μ*L fluoro-substrate peptide, 5 *μ*L NAD, 5 *μ*L developer, and 20 *μ*L sample was mixed thoroughly, and the fluorescence intensity was detected (ex. 350 nm, em. 450 nm) for 30 to 60 minutes at 1-2 min interval on a SpectraMax M5 system.

### 4.3. Western Blotting

Protein extracts from HUVECs or PMVECs were separated by SDS-PAGE and transferred to the PVDF membrane. The membrane was incubated with 5% BSA to block the nonspecific site for 1 h and incubated overnight at 4°C with primary antibodies for Sirt1, SOD2, *β*-catenin, acetyl-p53 (K319), p53, and 3-nitrotyrosine at 1 : 1000. After using a secondary antibody for incubation for 1 h, blots were visualized with the chemiluminescence method.

### 4.4. Measurement of Intracellular ROS Production

The level of ROS generation was assessed using peroxide-sensitive dye H_2_DCF-DA (10 *μ*M, 30 min), followed by PBS washes for three times. Fluorescence of cells was detected through fluorescent microscopy or BD FACSVerse flow cytometry [57].

### 4.5. Detection of Superoxide Dismutase

According to the manufacturer's protocol, cells were collected with 0.25% trypsin and washed with PBS three times. Afterwards, the contents of SOD were detected using a Total Superoxide Dismutase Assay Kit with WST-8 (S0101, Beyotime), followed by concentration determination using ELISA kits.

### 4.6. Measurement of TER

As previously described [58], 200 *μ*L of cells mounting to 10^5^/mL were seeded onto the transwell upper chamber. The monolayer cellular barrier property was measured through detecting the TER value across the confluent ECs with EVOM^2^ (World Precision Instrument, USA). The value of the measured monolayer was expressed by the formula: TER = TER of the experimental chamber minus TER of the cell-free chamber. Measurement was performed in triplicate and analyzed as percentage relative to TER level at time 0.

### 4.7. Dextran Transendothelial Flux

As previously described [59], cells were grown to a confluent monolayer and exposed to different stimulations. The tracer FITC-labelled dextran (1 *μ*g/mL) was added to the upper chamber to be incubated for 45 minutes. Later concentration of the dextran in the upper and bottom chamber was measured (ex. 488 nm, em. 525 nm) using an HST 7000 microplate reader.

### 4.8. Transfection of HUVECs with Sirt1 siRNA

HUVECs were cultured on 6-well plates until 30%–50% confluence and transfected with Sirt1 siRNA and control siRNA using Lipofectamine 2000 transfection according to the manufacturer's protocol. After 48–72 h, cells were exposed to different treatments.

### 4.9. Immunofluorescence Staining

HUVECs were cultured in confocal wells and subjected to different treatments. After cells were washed with PBS three times, they were fixed with 3.7% formaldehyde and perforated with 0.5% Triton X-100. Sequentially, ECs were blocked with 5% BSA to prevent nonspecific conjunction and incubated with a primary antibody against VE-cadherin at 4°C overnight, followed by incubation with an FITC-conjugated rabbit antibody. For F-actin staining, ECs were treated with rhodamine phalloidin at room temperature for 1 h. Cells were detected with a confocal scanning microscope (Zeiss, Germany) eventually.

### 4.10. PMVEC Isolation

As previously described [60], lungs of wild-type and RAGE^−/−^ mice were isolated, sliced, and digested in collagenase type I for 45 minutes, followed by filtration through 70 *μ*m nylon filters and centrifugation at 300 ×g for 10 minutes. Then, add appropriate CD31 to be incubated with the deposit and rotate thoroughly for 15 minutes. Afterwards, use the MACS separator to harvest the labelled cells.

### 4.11. LPS Treatment of Mice

Wild-type and RAGE^−/−^ C57 mice (18–20 g) were acquired from the lab center of Southern Medical University and Kanazawa University, respectively. The protocol using mice was approved by the Animal Care Committee of the Southern Medical University of China and was in accordance with the National Institutes of Health guidelines for ethical animal treatment. Mice were divided into four groups, including the saline group, LPS-treated group, SRT1720 + LPS-treated group, and LPS-treated RAGE^−/−^ group. Among these groups, mice in the LPS-treated group were treated with 15 mg/mL LPS via intraperitoneal injection. Mice in SRT1720 + LPS group were treated with 10 mg/kg SRT1720 via tail vein injection 2 h prior to LPS injection. Mice were anesthetized via intramuscular injection with 13.3% ethyl carbamate plus 0.5% chloralose (0.65 mL/kg) before carotid vein cannulation. The mice were placed on a platform to allow laparotomy incision and exposure of the appropriate mesenteric venules in the proximal ileum. Afterwards, 1 mL FITC-dextran (100 mg/mL) was administrated via the carotid vein firstly with 0.15 mg/kg/min dextran infusion constantly. Sequentially, fluorescence intensity was detected (ex. 488 nm, em. 525 nm), and the image was acquired in a fluorescence microscope (Nikon TE-300, Nikon Co., Japan). The formula calculating the permeability was as follows: △*I* = *I*_o_/*I*_i_, where *I*_o_ indicates the intensity inside the vessel while *I*_i_ refers to the intensity outside the vessel [61, 62].

### 4.12. Statistical Analysis

The significance of variability was analyzed by one-way ANOVA. All data were presented as mean ± SE from at least three dependent experiments. *P* < 0.05 was considered to be significant.

## Supplementary Material

Supplementary Figure 1. LPS and SRT1720 have no effect on total p53. Cells were treated with LPS or pretreated with SRT1720. Total p53 was examined using WB.
