# Supplementary material for: Sirt1 Protects Endothelial Cells against LPS-Induced Barrier Dysfunction
Source: Oxid Med Cell Longev. 2017 Oct 25;2017:4082102. doi: 10.1155/2017/4082102 (PMC5676476; doi:10.1155/2017/4082102)
Supplement: Supplementary file 1 — Supplementary Figure 1. LPS and SRT1720 have no effect on total p53. Cells were treated with LPS or pretreated with SRT1720. Total p53 was examined using WB. [file 4082102.f1.pptx]

## Slide 1
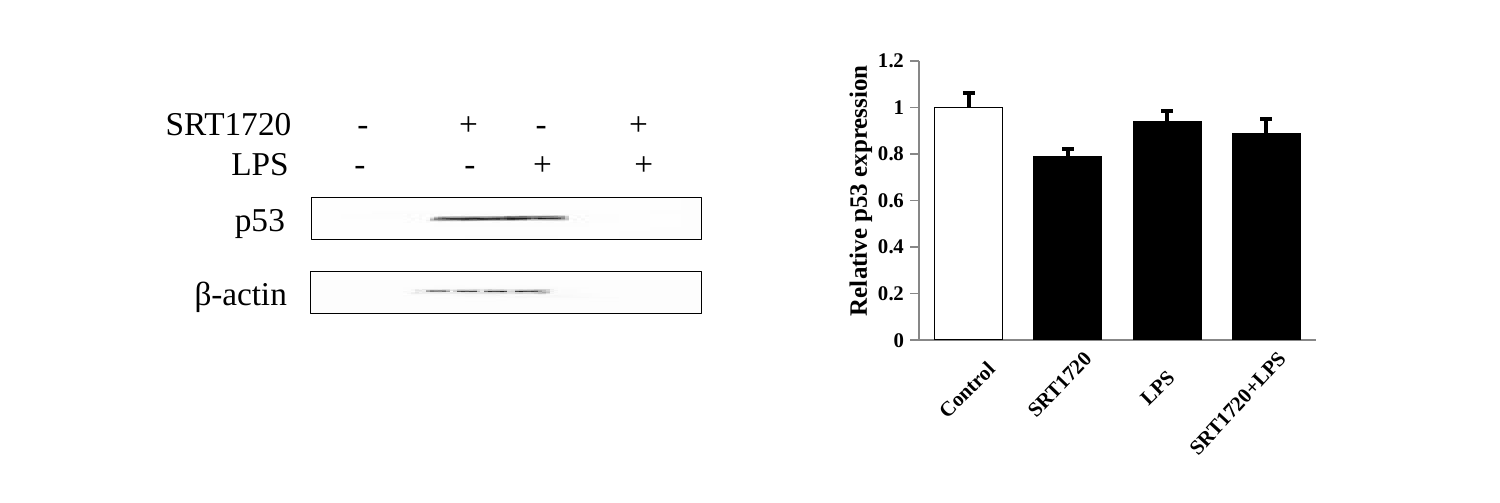

### Chart
| Category | wild type |
|---|---|
| Control | 1.0 |
| SRT1720 | 0.7867743 |
| LPS | 0.9406297 |
| SRT1720+lps | 0.8862902 |SRT1720 - + - +
 LPS - - + +
Relative p53 expression
 p53
β-actin
 SRT1720
Control
 LPS
SRT1720+LPS
